# Efficacy of Fecal Microbiota Transplantation in Irritable Bowel Syndrome: A Meta-Analysis of Randomized Controlled Trials

**DOI:** 10.3389/fcimb.2022.827395

**Published:** 2022-02-28

**Authors:** Jie Wu, Liang Lv, Chunlian Wang

**Affiliations:** Department of Gastroenterology, The Second Xiangya Hospital of Central South University, Changsha, China

**Keywords:** irritable bowel syndrome, fecal microbiota transplantation, intestinal microbiota, microbiota, meta-analysis

## Abstract

**Background:**

Randomized controlled trials (RCTs) have examined the efficacy of fecal microbiota transplantation (FMT) in irritable bowel syndrome (IBS) with inconsistent results. We performed a meta-analysis to assess both the short- and long-term efficacy of FMT in IBS.

**Methods:**

MEDLINE, EMBASE, and the Cochrane Central Register were searched through September 2021. RCTs recruiting adult patients with IBS that compared FMT with placebo with dichotomous data of response to therapy were eligible. Dichotomous data were pooled to obtain a relative risk (RR) of symptom not improving after therapy. RR was also pooled for adverse events (AEs). Continuous data were calculated using a mean difference for IBS-Quality of Life (IBS-QoL). GRADE methodology was used to assess quality of evidence.

**Results:**

The search strategy generated 658 citations. Seven RCTs comprising 472 patients with IBS were included. FMT was not associated with a significant improvement in global symptom in IBS at 12 weeks in comparison with placebo (RR 0.75, 95% CI 0.43–1.31) with high heterogeneity between studies (I^2^ 87%). Subgroup analyses showed that FMT was superior to placebo when administered *via* colonoscopy or gastroscope (RR 0.70, 95% CI 0.51–0.96; RR 0.37, 95% CI 0.14–0.99, respectively, while FMT was inferior to placebo when administered *via* oral capsules (RR 1.88, 95% CI 1.06–3.35). FMT induced a significant improvement in IBS-QoL compared to placebo (mean difference 9.39, 95% CI 3.86–14.91) at 12 weeks. No significant difference in the total number of AEs was observed between FMT and placebo (RR 1.20, 95% CI 0.59–2.47). FMT did not significantly improve global symptom in IBS at 1-year follow-up compared with placebo (RR 0.90, 95% CI 0.72–1.12). The GRADE quality evidence to support recommending FMT in IBS was very low.

**Conclusion:**

IBS patients may benefit from FMT when administered *via* colonoscopy or gastroscope. FMT may improve the quality of life of IBS. The long-term use of FMT in IBS warrants further investigation. There is very-low-quality evidence to support recommending FMT in IBS.

## 1 Introduction

Irritable bowel syndrome (IBS) is a common functional gastrointestinal disorder with a worldwide prevalence of 5%–10% (Ford et al., 2020; Oka et al., 2020), characterized by recurrent abdominal pain in association with defecation (Camilleri, 2021). IBS reduces the quality of life (QoL) and poses a high socioeconomic burden (Ford et al., 2020). The pathophysiology of IBS is heterogeneous and involves multiple factors including changes in microbiota, disturbed gut–brain axis, visceral hypersensitivity, increased permeability, and altered motility (Holtmann et al., 2016). IBS is classified into four subtypes according to the predominant stool type: diarrhea-predominant type (IBS-D), constipation-predominant type (IBS-C), mixed type (IBS-M), or unclassified type (IBS-U) (Mearin et al., 2016).

Alterations in gut microbiota in IBS patients compared to healthy controls have been well documented in various studies (Carroll et al., 2012; Tap et al., 2017; Pittayanon et al., 2019). IBS patients are also more likely to be linked to small intestinal bacterial overgrowth compared to healthy individuals (Shah et al., 2020). Furthermore, therapies targeting modulation of microbiota such as antibiotics, probiotics, and prebiotics have achieved promising effects in IBS patients (Ford et al., 2018).

Fecal microbiota transplantation (FMT) refers to the transfer of the intestinal microbiota from a healthy donor to a recipient (Vaughn et al., 2019). FMT has been proven to be a highly effective approach for recurrent *Clostridium difficile* infection (Quraishi et al., 2017; Rokkas et al., 2019). Recently, there has been emerging interest of utilizing FMT for the treatment of IBS. However, the results have been inconsistent. Two previous meta-analysis studies have assessed the efficacy of FMT in IBS in a short-term run (12 weeks), which included at most three full-text randomized controlled trials (RCTs) and two conference abstracts containing 267 subjects in total (Ianiro et al., 2019; Xu et al., 2019). Since then, there have been additional RCTs and more full-text articles have been published, allowing to explore more factors that may affect the efficacy of FMT for IBS. Additionally, none of the previous meta-analyses have evaluated the long-term efficacy of FMT for IBS as well as the impact of FMT on quality of life. A recent study systematically reviewed the published RCTs without conducting a meta-analysis (El-Salhy et al., 2021). We aimed to perform an up-to-date meta-analysis of RCTs to assess both the short- and long-term efficacy of FMT in IBS.

## 2 Methods

### 2.1 Search Strategy and Study Selection

We conducted the search using Medline, Embase, and the Cochrane Central Register of Controlled Trials to identify studies published from inception to September 2021. We manually searched clinicaltrials.gov for potential unpublished trials. Abstract books of conference proceedings including United European Gastroenterology Week, Digestive Disease Week, the American College of Gastroenterology Annual Meeting, the Asian Pacific Digestive Week, and the Federation of Neurogastroenterology & Motility conference between 2016 and 2021 were also hand-searched to identify studies that were published only in abstract form.

Studies on IBS were searched using “irritable bowel syndrome” (as a Mesh term), “irritable bowel syndrome,” “irritable colon,” “irritable bowel”, “fgids”, “functional gastrointestinal disorders”, and “IBS” (as free-text terms). Studies on FMT were searched using “fecal microbiota transplantation” (as a Mesh term), “microbiota transplantation*”, “intestinal microbiota transfer*”, “fecal transplantation*”, “donor feces infusion*”, “fecal microbiota transplantation”, and “FMT” (as free-text terms). We combined these two searches using the set operator AND. The detailed search strategy is provided in **Appendix S1**.

Randomized controlled trials were eligible if they met the following criteria: adults with IBS (participants aged ≥18 years); diagnosis of IBS either based on a clinician’s opinion or specific diagnostic criteria (Rome Criteria, Manning, etc.); minimum duration of 1-week follow-up after therapy; FMT compared with placebo; and dichotomous assessment of global symptom response (or no response) to therapy. Crossover RCTs in the first period were also eligible. These eligibility criteria were defined prospectively.

Two investigators (WJ and LL) independently searched the literature. There were no language restrictions. Foreign language papers were translated where required. Disagreements were resolved by discussion.

### 2.2 Outcome Assessment

The primary outcome was global symptom not improving in comparison between FMT and placebo at 8–12 weeks. Secondary outcomes included IBS-QoL, adverse events (AEs), and global symptom not improving at long-term follow-up.

### 2.3 Data Extraction

Two investigators (WJ and LL) independently extracted the data onto a Microsoft Excel Spreadsheet (Microsoft 365 Edition; Redmond, WA, USA) with efficacy (response or no response to therapy) and adverse events as dichotomous outcomes and IBS-QoL (mean, s.d., number of subjects) as a continuous outcome. Collected data for each study included the following: publication year, country of origin, study setting, IBS diagnosis criteria and subtype, methodology used in the study, primary and secondary endpoints, study population (female%), FMT and placebo administration, adverse events, and follow-up information. Data were extracted as intention-to-treat analyses with dropouts assumed to be treatment failures. We contacted the original authors for further information where necessary. Disagreements on extraction were resolved again by discussion.

### 2.4 Quality Assessment and Risk of Bias

We performed quality assessment for each study using the Cochrane Risk of Bias Tool (Higgins et al., 2011). We assessed the method used for randomization, whether allocation was concealed, whether blinding was implemented for participants and researchers, whether there was evidence of incomplete outcome assessment and selected reporting, and other sources of bias. Two independent investigators (WJ and LL) performed the quality assessment. Any disagreements were resolved by discussion.

### 2.5 GRADE Summary of Evidence

Grading of the evidence was done according to Grading of Recommendations Assessment Development and Evaluation (GRADE) methodology (Guyatt et al., 2011). Risk of bias, inconsistency, indirectness, imprecision, and potential publication bias were assessed.

### 2.6 Data Synthesis and Statistical Analysis

We pooled relative risks (RRs) with 95% confidence intervals (CIs) of remaining symptomatic after FMT compared to placebo. RRs were also used to assess AEs, where if the 95% CI did not cross 1, statistical significance is reached. A mean difference in IBS-QoL between FMT and placebo was calculated. Data were pooled with a random-effect model. We planned to assess for publication biases by Egger’s test with funnel plots where more than 10 studies were present (Sterne et al., 2011). Heterogeneity was evaluated with the I^2^ statistic, with values of <25%, 25% to 49%, 50% to 74%, and ≥75% considered no, low, moderate, or high levels of heterogeneity, respectively (Higgins et al., 2003). Where there were multiple intervention groups (e.g., different dosages used for the intervention group) in one study, it was recommended by the Cochrane Handbook (http://www.cochrane-handbook.org/) to combine the multiple intervention groups to create a single pair-wise comparison. Data analyses were performed using Review Manager (Version 5.4, RevMan for Windows, the Nordic Cochrane Centre, Copenhagen, Denmark) and STATA 15.1 (StataCorp, College Station, TX).

## 3 Results

**Figure 1:**
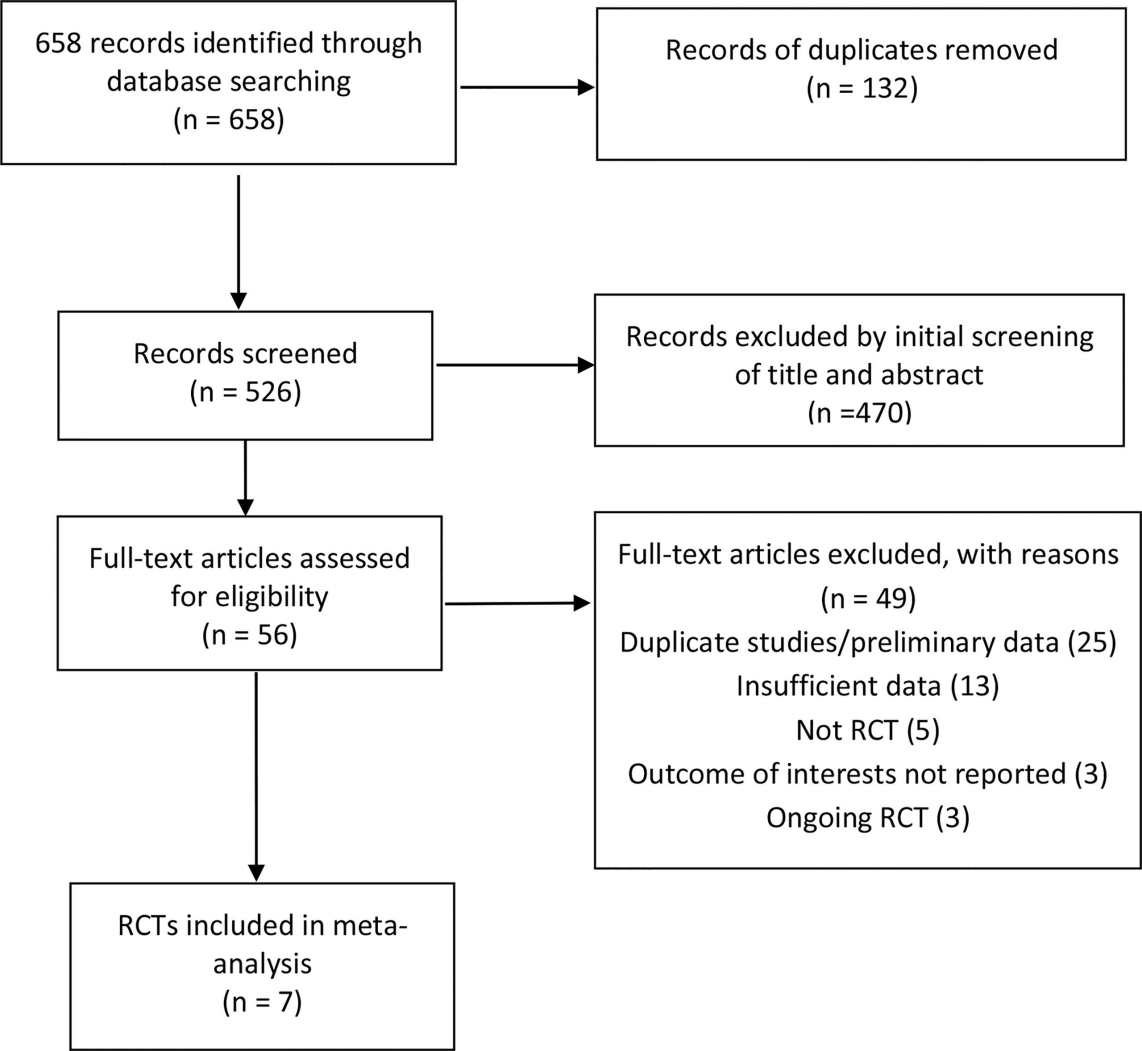
Flow diagram of included RCTs identified for meta-analysis.

The search strategy generated 658 citations, of which 56 citations were reviewed for full text after an initial screening of title and abstract. 49 studies were excluded for various reasons, leaving seven articles comprising seven RCTs eligible in the meta-analysis (**Figure 1**) (Halkjaer et al., 2018; Johnsen et al., 2018; Aroniadis et al., 2019; Holster et al., 2019; El-Salhy et al., 2020; Lahtinen et al., 2020; Holvoet et al., 2021). The seven RCTS were all full-text articles. The characteristics of included RCTs are shown in **Table 1**. A total of 472 IBS patients were included with female subjects accounting for 67.0%. Six studies were conducted in Europe (Halkjaer et al., 2018; Johnsen et al., 2018; Holster et al., 2019; El-Salhy et al., 2020; Lahtinen et al., 2020; Holvoet et al., 2021), and one in USA (Aroniadis et al., 2019). Four studies (Johnsen et al., 2018; Holster et al., 2019; El-Salhy et al., 2020; Holvoet et al., 2021) were performed in a single center, one study in two centers (Halkjaer et al., 2018), and two studies in three centers (Aroniadis et al., 2019; Lahtinen et al., 2020). Participants came from either primary or tertiary care. IBS was diagnosed by Rome III criteria in six studies (Halkjaer et al., 2018; Johnsen et al., 2018; Aroniadis et al., 2019; Holster et al., 2019; Lahtinen et al., 2020; Holvoet et al., 2021) and by Rome IV criteria in one study (El-Salhy et al., 2020). Three studies (Johnsen et al., 2018; Aroniadis et al., 2019; Holvoet et al., 2021) included only non-constipation IBS patients, and four studies (Halkjaer et al., 2018; Holster et al., 2019; El-Salhy et al., 2020; Lahtinen et al., 2020) recruited IBS patients of all three subtypes (IBS-C, IBS-D, IBS-M). Four RCTs (Halkjaer et al., 2018; Johnsen et al., 2018; Aroniadis et al., 2019; El-Salhy et al., 2020) recruited IBS patients with moderate to severe severity, and one RCT (Holvoet et al., 2021) recruited refractory IBS patients with predominant bloating, while the other two RCTs (Holster et al., 2019; Lahtinen et al., 2020) did not specify the severity information of IBS.

**Table 1:** Characteristics of included RCTs.

Author (year)	Country	Study setting	IBS criteria and subtype	Methodology	Primary endpoint	Secondary endpoints	Sample size (% female)	FMT intervention	Control intervention	Adverse events	Follow-up (responders/N)
Halkjaer et al. (2018)	Denmark	Two centers, tertiary care	Rome III, IBS-SSS ≥175, 33.3% IBS‐C, 29.4% IBS‐D, 37.3% IBS‐M	Computer-generated randomization with 1:1 allocation, in blocks, double-blinded	Decrease in IBS-SSS ≥50 points at 3 months	Change in IBS-QoL, microbiota profile	52 (67.3)	25 FMT capsules consisting of 50 g frozen donor stool daily × 12 d, from mixed samples of 4 donors	25 placebo capsules daily × 12 d	22/26(FMT), 15/26 (control)	6 months
Johnsen et al. (2018)	Norway	Single-center, primary care	ROME III, IBS-SSS ≥175, 53.0% IBS‐D, 47.0% IBS‐M	Computer-generated randomization with 2:1 allocation, in blocks, double-blinded; allocation in sealed opaque envelope	Decrease in IBS-SSS ≥75 points at 3 months	Decrease in IBS-SSS ≥75 points at 12 months	83 (66.3)	Single FMT consisting of 50–80 g both fresh and frozen (1:1) donor stool *via* colonoscopy, from mixed samples of 2 donors	50‐80 g autologous stool *via* colonoscopy	3/55 (FMT), 3/28(control)	12 months, 31/55 in FMT vs. 10/28 in control
Holster et al. (2019)	Sweden	Single-center, tertiary care	Rome III, 25.0% IBS‐C, 56.2% IBS‐D, 18.8% IBS‐M	Randomization with 1:1 allocation, double-blinded	Decrease in GSRS-IBS ≥30%	Change in IBS-QoL, IBS-SSS, depression, anxiety, barostat test, microbiota profile	16 (50)	Single FMT, consisting of 30 g fresh donor stool *via* colonoscopy, from single sample of either of the 2 donors	Single 30 g autologous stool *via* colonoscopy	4/8 (FMT), 7/19 (placebo)	6 months
Aroniadis et al. (2019)	USA	Three centers, primary and tertiary care	Rome III,IBS-SSS ≥175, 100% IBS-D	Computer-generated randomization with 1:1 allocation, in blocks, double-blinded cross-over; allocation in sealed envelope	Decrease in IBS-SSS ≥50 points at 12 weeks	Change in IBS-QoL, depression, anxiety, stool consistency, microbiota profile	48 (37.5)	25 FMT capsules consisting of 28 g frozen donor stool daily × 3 d, from single sample of either of the 4 donors	25 placebo capsules daily × 3 d	23/48 (FMT), 24/48 (control)	24 weeks
Lahtinen et al. (2020)	Finland	Three centers, primary and tertiary care	Rome III, 51.0% IBS‐D, 6.1% IBS‐C, 14.3% IBS‐M, 28.6% IBS-U	Randomization with 1:1 allocation, in blocks, double-blinded	Decrease in IBS-SSS≥ 50 points at 12 weeks	Change in IBS-QoL, depression, anxiety, stool consistency, microbiota profile	49 (59.2)	Single FMT consisting of 30g frozen donor stool *via* colonoscopy, from single donor	Single 30 g autologous stool *via* colonoscopy	7/23(FMT), 10/26(control)	52 weeks, 5/23 in FMT vs. 8/26 in control
El-Salhy et al. (2020)	Norway	Single center, tertiary care	Rome IV, IBS-SSS ≥175, 38.4% IBS-D, 37.8% IBS-C, 23.8% IBS-M	Computer-generated randomization with 1:1:1 allocation, in blocks, double-blinded	Decrease in IBS-SSS ≥ 50 points at 3 months	Change in IBS-QoL, dysbiosis index, microbiota profile	164 (81.1)	Single FMT consisting of 30 or 60 g donor frozen stool to the duodenum *via* gastroscopy, from one super donor	Single autologous stool *via* gastroscopy	48/55(FMT 30 g), 42/55 (FMT 60 g), 12/55 (control)	12 months, 32/55 in 30 g FMT, 35/55 in 60 g FMT
Holvoet et al. (2021)	Belgium	Single-center, tertiary care	Rome III, refractory IBS with severe bloating, IBS-D, IBS-M	Computer-generated randomization with 2:1 allocation, double-blinded, cross-over	Adequate relief of overall symptom at 12 weeks	Change in IBS- QoL, IBS symptom, stool consistency, microbiota profile	62 (61.3)	Single FMT consisting of donor fresh stool to the duodenum *via* nasojejunal tube from single sample of either of two donors	Single autologous stool *via* nasojejunal tube	NA	12 months, 5/43 in FMT vs. 0/19 in control

RCTs, randomized controlled trials; FMT, fecal microbiota transplantation; IBS, irritable bowel syndrome; IBS-D, IBS-C, IBS-M, IBS-U, IBS with diarrhea-predominant, constipation-predominant, mixed subtype, and IBS-unclassified; IBS-SSS, IBS-severity scoring system; IBS-QoL, IBS-Quality of Life; NA, not available.

FMT was administered *via* oral capsules in two RCTs (Halkjaer et al., 2018; Aroniadis et al., 2019), *via* colonoscopy in three RCTs (Johnsen et al., 2018; Holster et al., 2019; Lahtinen et al., 2020), and *via* gastroscope (or nasojejunal tube) to the duodenum in two RCTs (El-Salhy et al., 2020; Holvoet et al., 2021). Two RCTs used the FMT sample from mixed donors (Halkjaer et al., 2018; Johnsen et al., 2018), and the other four RCTs all used the sample from a single donor (Aroniadis et al., 2019; Holster et al., 2019; El-Salhy et al., 2020; Lahtinen et al., 2020; Holvoet et al., 2021). Fresh stool FMT was used in two studies (Holster et al., 2019; Holvoet et al., 2021), and frozen stool FMT in four studies (Halkjaer et al., 2018; Aroniadis et al., 2019; El-Salhy et al., 2020; Lahtinen et al., 2020), while FMT containing both fresh and frozen stool was used in only one study (Johnsen et al., 2018). In one study, FMT was performed at the doses of 30 and 60 g, respectively (El-Salhy et al., 2020). The follow-up of FMT ranged from 3 to 12 months.

### 3.1 Treatment Efficacy

#### 3.1.1 Failure to Achieve Symptom Improvement in IBS

**Figure 2:**
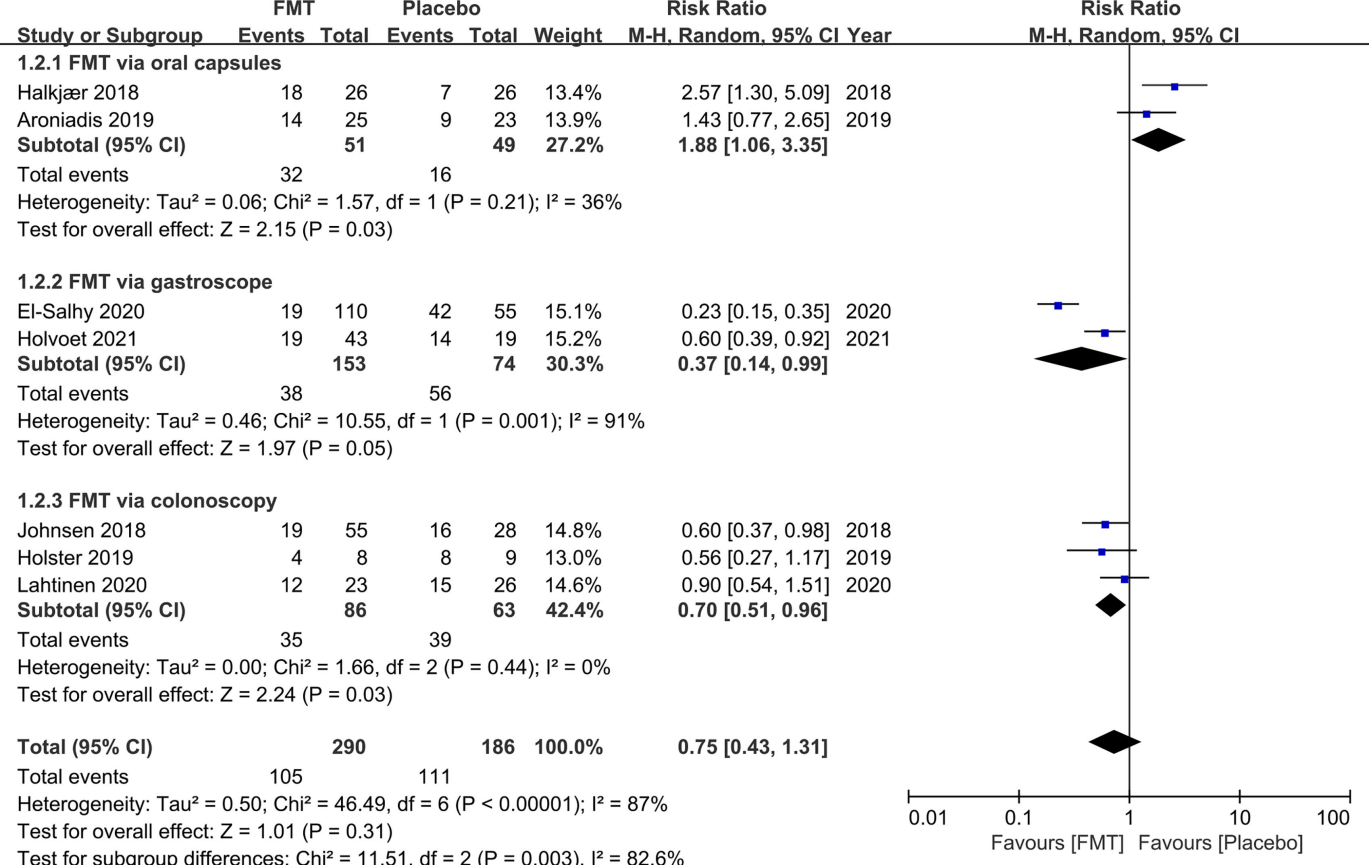
Forest plot of all included RCTs of global symptom not improving in comparison between FMT and placebo in irritable bowel syndrome at 12 weeks.

All seven RCTs provided dichotomous data of therapy response to FMT compared to placebo. The pooled response rate of FMT and placebo at week 12 was 55.0% (95% CI: 38.9%–71.1%) and 40.8% (95% CI: 26.0%–55.7%), respectively. FMT did not induce a significant reduction in global symptom at week 12 compared to placebo (pooled RR 0.75, 95% CI 0.43–1.31) when data were pooled from all seven RCTs, with high heterogeneity between studies (I^2^ 87%) (**Figure 2**). Only seven studies were included making it inadequate (less than 10) to assess for publication bias.

To explore the heterogeneity and factors that may affect the efficacy for FMT, we further did subgroup analyses according to the modality of FMT delivery, single or mixed donor sample, fresh or frozen donor stool, placebo type, study setting, IBS criteria, IBS subtype and severity, and FMT dosage (**Table 2**). FMT delivered *via* colonoscopy and gastroscope was superior to placebo when data were pooled from three RCTs and two RCTs, respectively (RR 0.70, 95% CI 0.51–0.96, I^2^ 0%; RR 0.37, 95% CI 0.14–0.99, I^2^ 91%, respectively). Conversely, pooling data from two RCTs, FMT was inferior to placebo when administered *via* oral capsules (RR 1.88, 95% CI 1.06–3.35) with low heterogeneity (I^2^ 36%).

**Table 2:** Subgroup analyses comparing FMT with placebo in IBS.

	No. of RCTs	RR	95% CI	I^2^
All RCTs	7	0.75	(0.43–1.31)	87%
*Route of delivery*				
**Oral capsules**	2	1.88	(1.06–3.35)	36%
**Colonoscopy**	3	0.70	(0.51–0.96)	0%
**Gastroscopy**	2	0.37	(0.14–0.99)	91%
*Mixed or single donor sample*				
Mixed	2	1.22	(0.30–5.04)	91%
Single	5	0.62	(0.33–1.17)	86%
*Stool type*				
Frozen stool	4	0.91	(0.31–2.68)	94%
**Fresh stool**	2	0.59	(0.41–0.85)	0%
Both	1	0.60	(0.37–0.98)	/
*Placebo type*				
**Inactive solution**	2	1.88	(1.06–3.35)	36%
**Autologous stool**	5	0.52	(0.32–0.86)	79%
*Center site*				
**Single center**	4	0.46	(0.27–0.77)	77%
Two centers	1	2.57	(1.30–5.09)	/
Three centers	2	1.10	(0.70–1.72)	21%
*Study setting*				
Primary care	1	0.60	(0.37–0.98)	/
Tertiary care	2	1.10	(0.70–1.72)	21%
Primary and tertiary care	4	0.65	(0.25–1.66)	92%
*IBS criteria*				
Rome III	6	0.91	(0.59–1.42)	73%
Rome IV	1	0.23	(0.15–0.35)	/
*IBS subtype*				
Non-constipation subtype	3	0.77	(0.47–1.28)	66%
All subtype	4	0.73	(0.26–2.04)	92%
*IBS severity*				
All	2	0.77	(0.49–1.20)	8%
Moderate to severe	4	0.82	(0.29–2.33)	93%
Refractory	1	0.60	(0.39–0.92)	/
*FMT dosage*				
≤30 g	2	0.77	(0.49–1.20)	8%
>30 g	4	0.82	(0.29–2.33)	93%
Not specified	1	0.60	(0.39–0.92)	/

Subgroup analyses reaching significance are highlighted in bold.

RCTs, randomized controlled trials; FMT, fecal microbiota transplantation; IBS, irritable bowel syndrome; RR, relative risk; CI, confidence interval.

Pooling data from two RCTs, IBS patients may benefit from FMT when fresh donor stool was used (RR 0.59, 95% CI 0.41–0.85) with no heterogeneity (I^2^ 0%). This effect was not seen in frozen stool FMT when data were pooled from four RCTs (RR 0.91, 95% CI 0.31–2.68) with high heterogeneity (I^2^ 94%).

Pooling data from four RCTs, FMT was superior to placebo when it was performed at a single center (RR 0.46, 95% CI 0.27–0.77) with high heterogeneity between studies (I^2^ 77%). FMT also showed efficacy over placebo when the autologous stool was used as control (RR 0.52, 95% CI 0.32–0.86), whereas an opposite effect was seen when an inactive solution was used as control (RR 1.88, 95% CI 1.06–3.35). There were no other significant results within the subgroup analyses.

#### 3.1.2 IBS-QoL

**Figure 3:**
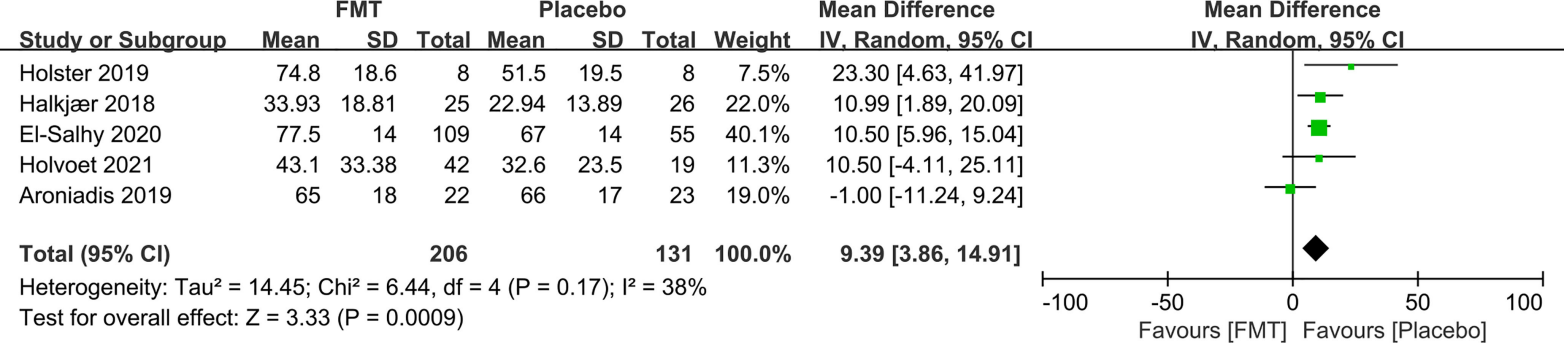
Forest plot of RCTs of quality of life in comparison between FMT and placebo in irritable bowel syndrome.

IBS-QoL was used in six RCTs. Three RCTs did not present the numerical values of IBS-QoL. We attempted to contact the original investigators, and one did not respond to our queries. Hence, the mean difference for IBS-QoL was only analyzed in five RCTs. FMT induced a significant improvement in quality of life of IBS at week 12 compared to placebo (mean difference 9.39, 95% CI 3.86–14.91) with low heterogeneity (I^2^ 38%) (**Figure 3**).

### 3.2 Adverse Events and Long-Term Follow-Up

AE data were available for five RCTs (Halkjaer et al., 2018; Johnsen et al., 2018; Aroniadis et al., 2019; El-Salhy et al., 2020; Holvoet et al., 2021). Overall, FMT was well tolerated, and no serious adverse events related to FMT were reported. Three serious AEs in three studies (one in each study) were reported, and all were considered irrelevant to FMT (Johnsen et al., 2018; Aroniadis et al., 2019; Holvoet et al., 2021). One patient reported transient nausea and vertigo after FMT, which was considered relevant to the colonoscopy procedure (Johnsen et al., 2018). The other two serious AEs that were reported in the control group were also irrelevant to FMT (Aroniadis et al., 2019; Holvoet et al., 2021).

**Figure 4:**
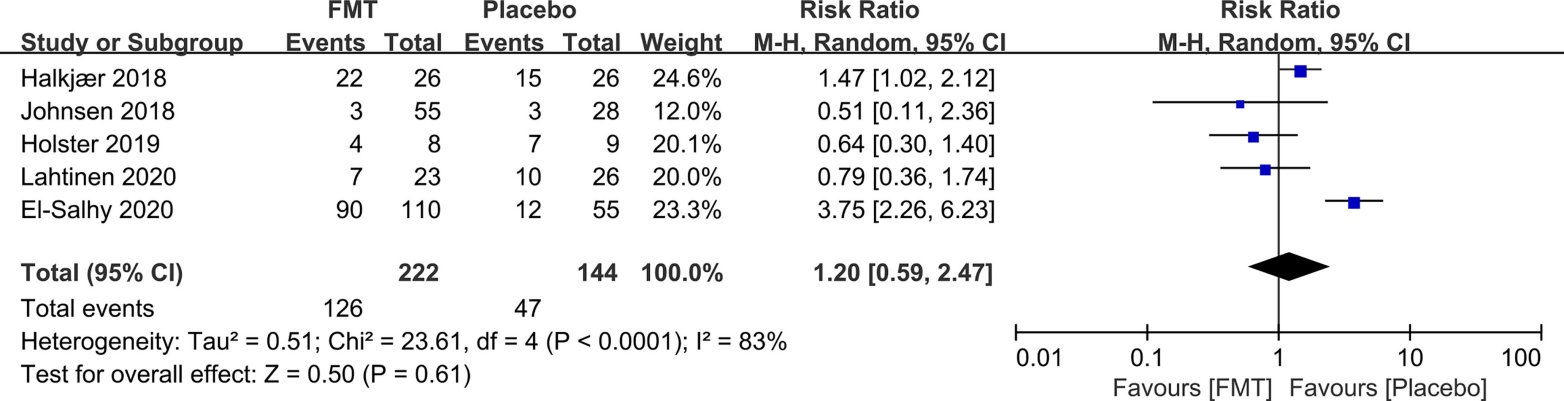
Forest plot of RCTs of adverse events in comparison between FMT and placebo in irritable bowel syndrome.

56.8% (126/222) of IBS patients reported AEs after receiving FMT, while 32.6% (47/144) reported AEs following placebo. However, there was no statistically significant difference in the total number of AEs between FMT and placebo (RR 1.20, 95% CI 0.59–2.47) with high heterogeneity (I^2^ 83%) (**Figure 4**). The AEs included abdominal pain, bloating, nausea, and diarrhea, and all were transient and self-resolved.

**Figure 5:**
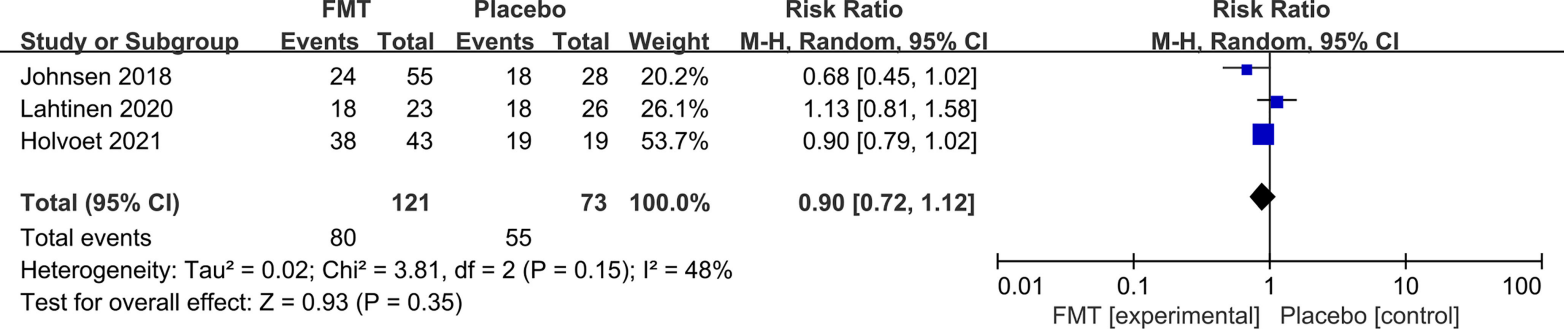
Forest plot of all included RCTs of global symptom not improving in comparison between FMT and placebo in irritable bowel syndrome at the 1-year follow-up.

Three RCTs reported the follow-up data of both FMT and control at 1 year. FMT was not associated with a significant global symptom improvement compared to placebo at the 1-year follow-up (RR 0.90, 95% CI 0.72–1.12) with low heterogeneity between studies (I^2^ 48%) (**Figure 5**).

### 3.3 Risk of Bias and GRADE Summary of Evidence

**Figure 6:**
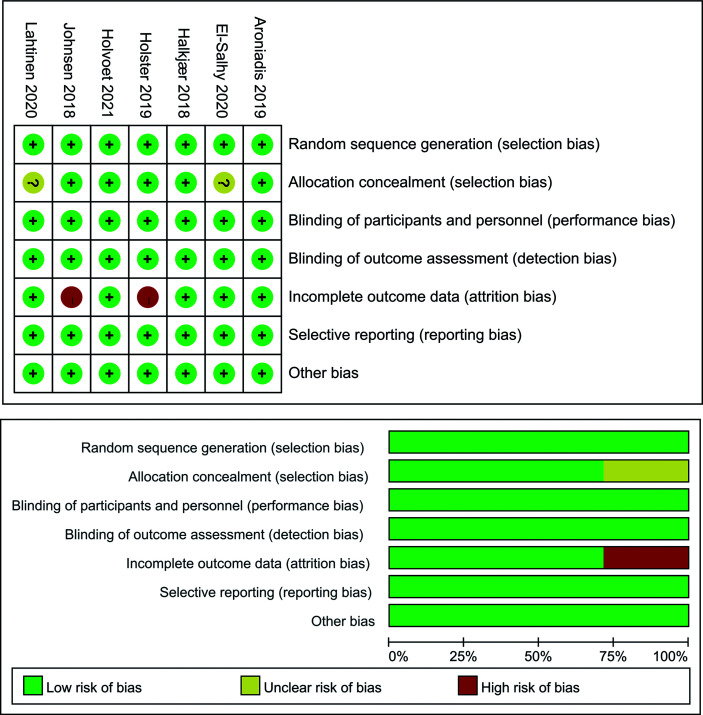
Risk-of-bias assessment of randomized controlled trials using Cochrane risk of bias tool.

**Figure 6** summarizes the risk of bias across studies using the Cochrane risk-of-bias tool. Three RCTs were regarded as low risk. Two RCTs were at high risk due to incomplete outcome data. Two RCTs were at unclear risk because of an unclear allocation method.

The overall quality of recommending FMT in IBS was “very low” according to GRADE criteria as most studies were at serious risk of bias; there was heterogeneity between studies; the imprecision was serious in the estimate of effect; publication bias was strongly suspected because RCTs in small sample sizes were included in the meta-analysis; and the conclusion may be changed when more future studies get published (**Table 3**).

**Table 3:** GRADE summary of evidence on the efficacy of FMT in IBS.

Certainty assessment							No. of patients		Effect		Certainty	Importance
No. of studies	Study design	Risk of bias	Inconsistency	Indirectness	Imprecision	Other considerations	FMT	Placebo	Relative (95% CI)	Absolute (95% CI)		
*IBS symptoms persist*												
7	RCTs	Serious	Very serious	Not serious	Serious	Publication bias strongly suspected	105/290 (36.2%)	111/186 (59.7%)	RR 0.75 (0.43 to 1.31)	149 fewer per 1,000 (from 340 fewer to 185 more)	⨁◯◯◯ VERY LOW	CRITICAL
*IBS symptoms persist: FMT via oral capsules*												
2	RCTs	Serious	Not serious	Not serious	Serious	Publication bias strongly suspected	32/51 (62.7%)	16/49 (32.7%)	RR 1.88 (1.06 to 3.35)	287 more per 1,000 (from 20 more to 767 more)	⨁◯◯◯ VERY LOW	CRITICAL
*IBS symptoms persist: FMT via gastroscope*												
2	RCTs	Serious	Not serious	Not serious	Serious	Publication bias strongly suspected	38/153 (24.8%)	56/74 (75.7%)	RR 0.37 (0.14 to 0.99)	477 fewer per 1,000 (from 651 fewer to 8 fewer)	⨁◯◯◯ VERY LOW	CRITICAL
*IBS symptoms persist: FMT via colonoscopy*												
3	RCTs	Serious	Not serious	Not serious	Serious	Publication bias strongly suspected	35/86 (40.7%)	39/63 (61.9%)	RR 0.70 (0.51 to 0.96)	186 fewer per 1,000 (from 303 fewer to 25 fewer)	⨁◯◯◯ VERY LOW	CRITICAL

FMT, fecal microbiota transplantation; RCTs, randomized controlled trials; CI, confidence interval; RR, risk ratio.

## 4 Discussion

We performed an exhaustive literature search and identified seven full-text published RCTs containing 472 patients with IBS and robustly summarized the contemporaneous evidence on both short- and long-term efficacy of FMT in IBS. Our up-to-date meta-analysis demonstrated that FMT was not associated with improvement in global symptom in IBS at 12 weeks when pooling data from all seven studies. There was high heterogeneity among studies. FMT was associated with an increased quality of life in IBS at week 12. No serious adverse events were related to FMT in IBS. No significant difference in the total number of adverse events was observed between FMT and placebo. FMT was not associated with global symptom improvement in IBS at the 1-year follow-up. The GRADE quality evidence to support recommending FMT in IBS was very low.

To explore the potential factors that may affect the efficacy of FMT in IBS including modality of FMT delivery, fresh or frozen stool FMT, single or mixed donor sample, placebo type, study setting, IBS criteria (Rome III or Rome IV), IBS subtype and severity, and FMT dosage, we performed extensive subgroup analyses. The subgroup analyses showed that IBS patients may benefit from FMT when administered *via* colonoscopy or gastroscope, or when fresh donor stool was used. The finding that IBS patients may benefit from FMT when studies were performed at a single canter in our meta-analysis may be attributed to the fact that FMT was administered either *via* colonoscopy or gastroscope in these studies.

Our meta-analysis demonstrated that IBS patients may benefit from FMT at 12 weeks when administered *via* colonoscopy or gastroscope, but not *via* oral capsules. FMT is commonly delivered by three modalities: oral capsules, gastroscope or nasojejunal probe to the duodenum, and colonoscopy. Oral capsule is well tolerated and the most acceptable way by participants, while colonoscopy requires bowel lavage and is time consuming. A national FMT registry in USA involving 259 participants who underwent FMT for treatment of *Clostridium difficile* infection showed that 85% of the FMT procedures were performed *via* colonoscopy (Kelly et al., 2021). The efficacy of FMT may vary between conditions when administered by different routes. One RCT compared FMT for the prevention of recurrent *Clostridium difficile* infection *via* oral capsules and colonoscopy, and both delivery ways exhibited high success rates of more than 95%. Nevertheless, subjects receiving oral capsules were more satisfied with the FMT experience compared with those administered *via* colonoscopy (Kao et al., 2017). FMT also showed promising effect in the treatment of active ulcerative colitis when administered *via* colonoscopy (Paramsothy et al., 2017; Costello et al., 2019). The use of FMT for obese patients also raised considerable interest, but the current evidence to support its use may be limited as a recent RCT did not find FMT to reduce body mass index in 22 patients (Allegretti et al., 2020).

Frozen stool FMT is as effective as fresh stool FMT in the treatment of recurrent *Clostridium difficile* infection (Lee et al., 2016; Tang et al., 2017). Compared to fresh stool FMT, frozen stool FMT costs less and is more convenient in clinical practice. We demonstrated that IBS patients may benefit from FMT when fresh donor stool was used. However, interpretation of this result should be very cautious as fresh stool FMT data were pooled from only two RCTs, where FMT was administrated *via* colonoscopy and nasojejunal tube, which may confound the result. There was high heterogeneity (94%) among studies using frozen FMT, making it far away to conclude that frozen FMT is not efficacious in IBS. Further research is needed to clarify whether FMT using fresh or frozen donor stool may affect the treatment efficacy for IBS.

IBS has a substantial impact on the quality of life of individuals concerning reduced work productivity and increased healthcare utilization (Oka et al., 2020). In a previous meta-analysis study, IBS-QoL was not pooled due to insufficient data (Xu et al., 2019). Our meta-analysis showed that FMT may improve the quality of life of IBS at week 12. Implications of this finding need further research.

Regarding the long-term efficacy of FMT, we demonstrated that FMT was not associated with global symptom improvement at the 1-year follow up. IBS is a chronic condition with symptom fluctuating and relapsing over time (Mearin et al., 2016). This may explain why single FMT may not achieve a long-lasting effect on IBS. Holvoet et al. (Holvoet et al., 2021) found that a second FMT showed efficacy in 67% of IBS patients who had an initial response to the first FMT, indicating that repeated FMT might be a long-term treatment option for IBS.

The underlying mechanism of FMT in IBS is still unclear. The efficacy of FMT in IBS may also be affected by many other factors, including donor selection, placebo response rate, study protocol, and heterogeneity of IBS. The selection of donor may play an important role in the efficacy of FMT. In the study by El-Salhy et al., FMT with the selection of a super donor induced a high responding rate of over 76% in IBS (El-Salhy et al., 2020). IBS patients also tend to have high placebo response rates. The pooled placebo response rate was 40.8% in our meta-analysis and was higher than the pooled placebo response rate of 27.3% in pharmacological RCTs of IBS (Bosman et al., 2021), which may also affect the efficacy of FMT to some extent.

Our study has limitations. *First*, we crudely performed the subgroup analysis of FMT dosage by simply dividing FMT into ≥30- and <30-g groups, which could not reflect the true dose response effect of FMT. El-Salhy et al. (El-Salhy et al., 2020) found that 60 g FMT induced a higher (but not statistically significant) response rate in IBS than 30 g FMT (89.1% vs. 76.9%). Further studies on the dose effect of FMT are needed. *Second*, heterogeneity persisted in some instances even after we did extensive subgroup analyses, which may be due to the variation in characteristics of studies, including different regions and clinical settings, donor selection, administration of FMT delivery, and the study protocol. *Third*, we combined IBS-D and IBS-M patients into one group as non-constipation IBS in the subgroup analysis. There might be putative differences between different IBS subtypes. Our meta-analysis was unable to examine this issue due to insufficient data provided in the included studies.

In conclusion, our up-to-date meta-analysis of RCTs demonstrated that IBS patients may benefit from FMT when administered *via* colonoscopy or gastroscope. FMT may improve the quality of life in IBS. FMT is overall safe in IBS. The long-term use of FMT in IBS warrants further investigation. The GRADE quality evidence to support recommending FMT in IBS was very low. Future RCTs may focus on investigating the potential factors that may affect the efficacy of FMT for IBS, including the delivery way of FMT, fresh or frozen stool FMT, FMT dosage, and specific IBS subtype. Replicate studies in large sample sizes with sufficient power are also needed to corroborate the findings from previous RCTs.

## Data Availability Statement

The original contributions presented in the study are included in the article/**Supplementary Material**. Further inquiries can be directed to the corresponding author.

## Ethics Statement

Ethical review and approval were not required for the study on human participants in accordance with the local legislation and institutional requirements. Written informed consent for participation was not required for this study in accordance with the national legislation and the institutional requirements.

## Author Contributions

JW conceived the study. JW and LL extracted and analyzed the data. JW, LL, and CW interpreted the data. The manuscript was written by JW and critically revised by LL and CW. All authors contributed to the article and approved the submitted version.

## Conflict of Interest

The authors declare that the research was conducted in the absence of any commercial or financial relationships that could be construed as a potential conflict of interest.

## Publisher’s Note

All claims expressed in this article are solely those of the authors and do not necessarily represent those of their affiliated organizations, or those of the publisher, the editors and the reviewers. Any product that may be evaluated in this article, or claim that may be made by its manufacturer, is not guaranteed or endorsed by the publisher.
