# Supplementary material for: Efficacy of Fecal Microbiota Transplantation in Irritable Bowel Syndrome: A Meta-Analysis of Randomized Controlled Trials
Source: Front Cell Infect Microbiol. 2022 Feb 28;12:827395. doi: 10.3389/fcimb.2022.827395 (PMC8919053; doi:10.3389/fcimb.2022.827395)
Supplement: Supplementary file 1 [file DataSheet_1.pdf]

Search Strategy

PubMed

#1 "Irritable Bowel Syndrome"[Mesh] OR (irritable colon) OR (irritable bowel syndrome) OR (irritable bowel) OR (gids) OR (functional gastrointestinal disorders) OR (IBS)

#2 "Fecal Microbiota Transplantation"[Mesh] OR (Microbiota Transplantation*, Fecal) OR (Transplantation*, Fecal Microbiota) OR (Intestinal Microbiota Transfer*) OR (Microbiota Transfer*, Intestinal) OR (Transfer*, Intestinal Microbiota) OR (Fecal Transplantation*) OR (Transplantation*, Fecal) OR (Fecal Transplant*) OR (Transplant*, Fecal) OR (Donor Feces Infusion*) OR (Feces Infusion*, Donor) OR (Infusion*, Donor Feces) OR (FMT)

#3 randomized controlled trial [pt]

#4 controlled clinical trial [pt]

#5 randomized [tiab]

#6 placebo [tiab]

#7 clinical trials as topic [mesh: noexp]

#8 randomly [tiab]

#9 trial [ti]

#10 #3 OR #4 OR #5 OR #6OR #7 OR #8 OR #9

#11 animals [mh] NOT humans [mh]

#12 #10 NOT #11

#13 #1 AND #2 AND #12

Embase

#1 'irritable colon'/exp OR 'irritable bowel syndrome*':ab,ti OR 'irritable bowel':ab,ti OR 'fgids':ab,ti OR 'functional gastrointestinal disorders':ab,ti OR 'ibs':ab,ti

#2 ‘Fecal Microbiota Transplantation’/exp OR (Microbiota Transplantation*, Fecal) OR (Transplantation*, Fecal Microbiota) OR (Intestinal Microbiota Transfer*) OR (Microbiota Transfer*, Intestinal) OR (Transfer*, Intestinal Microbiota) OR (Fecal Transplantation*) OR (Transplantation*, Fecal) OR (Fecal Transplant*) OR (Transplant*, Fecal) OR (Donor Feces Infusion*) OR (Feces Infusion*, Donor) OR (Infusion*, Donor Feces) OR (FMT)

#3 'clinical trial'/de OR 'randomized controlled trial'/de OR 'randomization'/de OR 'single blind procedure'/de OR 'double blind procedure'/de OR 'crossover procedure'/de OR 'placebo'/de OR 'prospective study'/de OR 'randomi?ed controlled' NEXT/1 trial* OR rct OR 'randomly allocated' OR 'allocated randomly' OR 'random allocation' OR allocated NEAR/2 random OR single NEXT/1 blind* OR double NEXT/1 blind* OR (treble OR triple) NEAR/1 blind* OR placebo*

#4 #1 AND #2 AND #3

Cochrane central register of controlled trials

#1 Irritable Bowel Syndrome: ME

#2 irritable bowel*

#3 #1 or #2

#4 (Fecal Microbiota Transplantation): ME

#5 (Microbiota Transplantation*, Fecal) OR (Transplantation*, Fecal Microbiota) OR (Intestinal Microbiota Transfer*) OR (Microbiota Transfer*, Intestinal) OR (Transfer*, Intestinal Microbiota) OR (Fecal Transplantation*) OR (Transplantation*, Fecal) OR (Fecal Transplant*) OR (Transplant*, Fecal) OR (Donor Feces Infusion*) OR (Feces Infusion*, Donor) OR (Infusion*, Donor Feces) OR (FMT)

#6 #4 OR #5

#7 #3 AND #6
